# Physician tobacco screening and advice to quit among U.S. adolescents – National Survey on Drug Use and Health, 2013

**DOI:** 10.1186/s12971-016-0107-6

**Published:** 2017-01-10

**Authors:** Lauren Collins, Sabrina L. Smiley, Rakiya A. Moore, Amanda L. Graham, Andrea C. Villanti

**Affiliations:** 1 Schroeder Institute for Tobacco Research and Policy Studies at Truth Initiative, 900 G Street, NW, Fourth Floor, Washington, DC USA; 2 Evaluation Science and Research at Truth Initiative, Washington, DC USA; 3 Department of Oncology, Georgetown University Medical Center/Cancer Prevention and Control Program, Lombardi Comprehensive Cancer Center, Washington, DC USA; 4 Department of Health, Behavior and Society, Johns Hopkins Bloomberg School of Public Health, Baltimore, MD USA

**Keywords:** Adolescent, Smoking, Smoking cessation, Physicians, United States

## Abstract

**Background:**

Initiating tobacco use in adolescence increases the risk of nicotine dependence and continued smoking. Physician screening for tobacco use increases the odds of physicians intervening with patients who smoke; However, without appropriate follow-through by the physician, screening for tobacco use is not enough to significantly increase cessation rates. Given the critical phase of development adolescence poses in tobacco use and evidence that physician intervention improves adult cessation efforts, we sought to examine physician tobacco use screening and advice to quit among adolescents (12–17 years).

**Methods:**

Using data from the 2013 National Survey on Drug Use and Health (NSDUH), we examined the prevalence and correlates of tobacco use screening in adolescent respondents who reported visiting their physician within the past year (*N* = 12,798). Multivariable logistic regression analyses explored the relationship between tobacco use screening and physician advice to quit in a sub-set of the sample who reported on physician advice to quit (*n* = 1,868), controlling for sociodemographics, cigarette use, and substance use and screening.

**Results:**

Only 49% of adolescents who visited a physician within the past year reported being screened for tobacco use. Adolescents who were screened by their physician were predominantly female (56.6%), White (60.1%), in late adolescence (83.0%), and covered by private health insurance (63.8%). Screening for tobacco use was highly correlated with physician advice to quit smoking, controlling for sociodemographic characteristics and cigarette use; this relationship was attenuated, but remained significant, after screening for alcohol and marijuana were added to the model. Hispanic adolescents were significantly less likely to receive physician advice to quit in all multivariable models.

**Conclusions:**

Our findings suggest missed opportunities for youth tobacco use prevention and cessation efforts in the clinical setting. Further research is needed to better facilitate an open dialogue on tobacco use between physicians and their adolescent patients.

## Background

Adolescence marks a developmental period in which there are increases in experimentation [1] and regular use of tobacco [2]. An estimated 5,700 people per day initiated smoking cigarettes in 2013 [3]. Adolescents (12–17 years) accounted for half of all initiates in that year, and 7.8% of adolescents reported past 30-day tobacco product use [3]. Initiating tobacco use in adolescence increases the risk of nicotine dependence and continued smoking [4–7]. Thus, tobacco prevention and cessation interventions may have a greater impact in adolescence than in adulthood when risk behaviors are more established. However, the proportion of past-year quit attempts among adolescent smokers decreased from 57.4% in 2001 to 48% in 2013 [8] suggesting that tobacco cessation efforts are not effectively reaching adolescent smokers.

Systematic reviews and meta-analyses of tobacco cessation interventions show that the more successful interventions consist of physicians and/or non-physicians engaged in one-on-one interactions as a means of tailoring efforts to the specific cessation needs of the smoker [9–11]. This individualized approach starts with identifying an individual’s smoking status. Screening patients for tobacco use allows physicians to identify the appropriate intervention - promoting quitting among current smokers, preventing relapse in former smokers, or encouraging never smokers to continue abstinence [12].

Physician screening for tobacco use increases the odds three-fold of physicians intervening with patients who smoke; However, without appropriate follow through by the physician, screening for tobacco use is not enough to significantly increase cessation rates [12]. The 2008 update to the U.S. Public Health Service Clinical Practice Guideline for Treating Tobacco Use and Dependence found it essential that physicians identify the tobacco use status of patients and intervene with all tobacco users identified [12]. Even brief physician advice lasting less than 3 min has resulted in a significant increase in quit rates among adult smokers compared to those who received no advice [12, 13].

Despite evidence supporting physician-based tobacco prevention and cessation interventions in adolescents, data from the 2011 National Youth Tobacco Survey shows only 20.8% of middle and high school students who visited a physician in the past 12 months were both screened for tobacco use and advised to quit [14]. Evidence suggests the rates of physician screening and advice to quit may differ among particular racial and ethnic groups. Data from the Memphis Health Project, a longitudinal study of smoking in adolescents in the mid-South, found that in a primarily African American sample of 11^th^ graders (82.9%), 28.9% of adolescents sampled were both screened for tobacco use and advised to quit by a physician [15].

To our knowledge, there is no recent data available on physician screening for tobacco use and advice to quit in a nationally representative sample of adolescents. Given the critical phase of development adolescence poses in tobacco use, and evidence that face-to-face interventions can improve smoking outcomes, this study used data from a recent national survey to examine physician tobacco use screening and advice to quit among adolescents who reported visiting a physician in the past 12 months.

## Methods

### Subjects and data collection

The National Survey on Drug Use and Health (NSDUH) is the primary source for population estimates of substance use and health-related behaviors among the U.S. general population. A detailed description of the survey methodology is reported elsewhere [3]. Briefly, the NSDUH uses a state-based design with an independent, multistage area probability sample within each state and the District of Columbia to produce a nationally representative sample of 67,838 individuals aged 12 years or older. Data from the 2013 NSDUH were used to examine the prevalence and correlates of tobacco use screening in adolescent respondents (ages 12–17) who reported visiting their physician in the past year (*N* = 13,034). For the purpose of these analyses, only those who responded to questions about physician screening were included, reducing the sample to 12,798. A sub-set of this sample who responded to questions about both physician screening and advice to quit were used to examine the relationship between physician tobacco use screening and physician provided advice to quit (*n* = 1,868).

## Measures

### Sociodemographic characteristics

Participants reported sex, age, race/ethnicity, and current school enrollment. Age was dichotomized as 12–13 years and 14–17 years to examine differences between early and late adolescence. Research has shown that an individual’s income and health insurance coverage impact access to care and the quality of care received [16, 17]. To examine how these factors may affect physician screening and advice to quit, participants’ parents served as proxies to collect information related to income (total family income, receipt of public assistance, receipt of food stamps) and health insurance.

### Tobacco and substance use

Participants were classified in three mutually-exclusive groups: never cigarette users, ever cigarette users (but not in the past 30 days), or past 30-day cigarette users. Never cigarette users were defined as those who did not report having ever smoked part or all of a cigarette. Ever cigarette users were defined as those who reported having ever smoked part or all of a cigarette, but not in the past 30-days. Past 30-day cigarette use was defined as having smoked part or all of a cigarette on at least one day in the past 30 days. Additional substance use included past 30-day alcohol and marijuana use, which were defined as having any alcohol or marijuana on at least one day in the past 30 days, respectively.

### Physician screening

Participants were asked one question in regards to physician screening for tobacco use: “During the past 12 months, did any doctor or other health care professional ask, either in person or on a form, if you use tobacco?” Participants who refused to respond or responded “don’t know” were excluded from analysis (*n* = 236). As it is recommended that physicians screen for all substance use and publicly available screening tools address multiple substances in the same screener [18], screening for alcohol and drug use were also examined. Participants were asked the same question as it relates to alcohol use and drug use.

### Physician advice to quit

The main outcome considered was whether or not adolescents received advice to quit from their physician. Advice to quit was assessed with the following question: “During the past 12 months, did any doctor or other health care professional advise you to quit smoking cigarettes or quit using any other tobacco products?”.

### Data analysis

All analyses were performed using Stata SE13.1 (StataCorp, 2013) and post-stratification weights were used to offset any non-response or non-coverage bias and produce nationally representative estimates. Missing data were handled with listwise deletion per Stata’s survey procedures. Bivariate analyses examined the prevalence of physician screening for tobacco use by sociodemographic characteristics, cigarette use, alcohol use, marijuana use, and screening for alcohol and marijuana use. Four nested multivariable logistic regression analyses explored the relationship between tobacco use screening and physician advice to quit, controlling for sociodemographic characteristics in Model A, followed by the addition of cigarette use in Model B, alcohol use and screening in Model C, and marijuana use and screening in Model D.

## Results

### Sample characteristics

Among the adolescents who completed the survey (*N* = 12,798), 51.7% were female (Table 1). More than two-thirds (71.7%) of the participants were between the ages of 14 and 17 years old. More than half of adolescents in the sample were non-Hispanic White (55.5%), 21.9% were Hispanic, 13.8% were non-Hispanic Black, and 5.1% were non-Hispanic Asian. Approximately 37% of participants had a total family income of $75,000 or more and more than half of adolescents (59.6%) had private medical insurance.

**Table 1:** Descriptive statistics of adolescents by physician tobacco use screening; NSDUH 2013 (*n* = 12,798)

	Overall	Physician Screened for Tobacco Use		
		Yes	No	*P*
		*n* = 6324 (49.4%)	*n* = 6474 (50.6%)	
Sex, n (%)				<.001
Male	6252 (48.3)	2810 (43.4)	3442 (52.7)	
Female	6546 (51.7)	3514 (56.6)	3032 (47.2)	
Age, n (%)				<.001
12–13 yo	3641 (28.3)	1100 (17.1)	2541 (38.8)	
14–17 yo	9157 (71.7)	5224 (83.0)	3933 (61.2)	
Race/Ethnicity, n (%)				<.001
Non-Hispanic White	7255 (55.5)	3872 (60.1)	3383 (51.3)	
Non-Hispanic Black	1780 (13.8)	788 (12.1)	992 (15.3)	
Hispanic	2449 (21.9)	1089 (20.5)	1360 (23.1)	
Non-Hispanic Asian	476 (5.1)	191 (3.8)	285 (6.3)	
Non-Hispanic Other	838 (3.8)	384 (3.5)	454 (4.1)	
Enrolled in School, n (%)	12683 (99.4)	6262 (99.3)	6421 (99.4)	ns
Total Family Income, n (%)				<.001
Less than $20,000	2292 (17.5)	1035 (16.2)	1257 (18.7)	
$20,000–$49,999	3751 (28.6)	1791 (27.1)	1960 (30.0)	
$50,000–$74,999	2236 (16.5)	1089 (16.4)	1147 (16.6)	
$75,000 or more	4519 (37.3)	2409 (40.3)	2110 (34.6)	
Receives Public Assistance, n (%)	536 (4.0)	237 (3.7)	299 (4.4)	<.001
Receives Food Stamps, n (%)	3003 (22.3)	1,362 (20.4)	1641 (24.0)	.002
Private Health Coverage, n (%)	7620 (59.6)	3973 (63.8)	3647 (55.7)	<.001
Cigarette Use				<.001
Never Use	10565 (84.2)	5063 (81.7)	5502 (86.4)	
Ever Use, No Current Use	1405 (10.3)	719 (11.1)	686 (9.5)	
Current Use (Past 30-Day)	828 (5.6)	542 (7.1)	286 (4.2)	
Alcohol, n (%)				
Past 30-Day Use	1576 (11.5)	923 (13.5)	653 (9.7)	<.001
Screened for Use	5,592 (42.4)	5,187 (82.6)	405 (5.3)	<.001
Marijuana, n (%)				
Past 30-Day Use	1013 (6.9)	622 (8.8)	391 (5.2)	<.001
Screened for Use	4,702 (35.4)	4,339 (68.3)	363 (5.0)	<.001

Cell counts reported are unweighted, but the percentages represent the weighted sample. All percentages reported are column percents

Overall, the majority of adolescents reported never using cigarettes (84.2%). Approximately 10% of adolescents had ever used a cigarette, with 5.6% reporting past 30-day cigarette use. Past 30-day alcohol use (11.5%) was more common than past 30-day marijuana use (6.9%) among this sample of adolescents.

### Physician screening for tobacco use

Fewer than half of adolescents who visited a physician in the past 12 months were screened for tobacco use (49.4%; Table 1). Adolescents who were screened for tobacco use by their physician were more likely to be female (56.6%), non-Hispanic White (60.1%), in late adolescence (83.0%), and covered by private health insurance (63.8%; all *p* values < .001). Adolescents who were screened for tobacco use were also more likely to be screened for alcohol use and drug use (82.6 and 68.3%, respectively; *p* values < .001).

### Physician advice to quit

Among the adolescents who reported on physician advice to quit (*n* = 1,868), the majority were past 30-day (43.2%; Table 2) and ever smokers (38.5%), followed by never (18.3%) users. Regardless of cigarette use status, the majority of adolescents were neither screened for nor advised to quit tobacco. More than one-third of ever users (37.7%) and past 30-day users (36.5%) were screened for tobacco use, but did not receive any advice to quit. Fewer than one-third of past 30-day users were screened for tobacco use and received advice to quit from their physician (26.3%).

**Table 2:** Physician interaction by adolescent’s smoking status; NSDUH 2013 (*n* = 1,868)

	Cigarette Use, n (%)		
	Never Use 342 (18.3)	Ever Use, Not Past 30 -Day 720 (38.5)	Past 30-Day Use 806 (43.2)
Neither Screened nor Advised	148 (46.4)	351 (48.2)	266 (37.3)
Only Screened	152 (42.4)	261 (37.7)	302 (36.5)
Screened and Advised	42 (11.1)	108 (14.2)	238 (26.3)

Cell counts reported are unweighted, but the percentages represent the weighted sample. All percentages reported are row percents

**Table 3:** Multivariable logistic regression models of physician advice to quit smoking among adolescents; NSDUH 2013 (*n* = 1,886)

	Model A		Model B		Model C		Model D	
Variable	OR (95% CI)	*p*	OR (95% CI)	*p*	OR (95% CI)	*p*	OR (95% CI)	*p*
Screened for Tobacco	12.60 (7.98, 19.88)	<.001	12.73 (8.15, 19.89)	<.001	7.12 (3.98, 12.74)	<.001	6.01 (3.35, 10.79)	<.001
Sex								
Female (ref)	–	–	–	–	–	–	–	–
Male	1.32 (.94, 1.86)	ns	1.50 (1.07, 2.12)	.021	1.67 (1.19, 2.36)	.004	1.60 (1.13, 2.26)	.009
Age								
12–13 (ref)	–	–	–	–	–	–-	–	–
14–17	.97 (.41, 2.32)	ns	.90 (.38, 2.15)	ns	.79 (.34, 1.84)	ns	.78 (.35, 1.70)	ns
Race								
Non-Hispanic White (ref)	–	–	–	–	–	–	–	–
Non-Hispanic Black	.74 (.39, 1.38)	ns	.81 (.43, 1.53)	ns	1.00 (.53, 1.90)	ns	.95 (.50, 1.79)	ns
Hispanic	.49 (.31, .76)	.002	.48 (.30, .76)	.002	.51 (.31, .82)	.006	.47 (.28, .78)	.004
Non-Hispanic Asian	.28 (.03, 2.27)	ns	.24 (.03, 1.98)	ns	.28 (.03, 2.37)	ns	.31 (.04, 2.35)	ns
Non-Hispanic Other	.84 (.50, 1.41)	ns	.88 (.52, 1.50)	ns	.79 (.44, 1.41)	ns	.75 (.40, 1.41)	ns
Family Income								
$20,001–$49,999 (ref)	–	–	–	–	–	–	–	–
Less than $20,000	.97 (.67, 1.40)	ns	.98 (.67, 1.42)	ns	.99 (.67, 1.47)	ns	1.05 (.69, 1.57)	ns
$50,000–$74,999	.93 (.55, 1.55)	ns	.91 (.53, 1.56)	ns	1.00 (.58, 1.72)	ns	1.03 (.60, 1.77)	ns
$75,000 or More	1.01 (.63, 1.59)	ns	1.06 (.65, 1.70)	ns	1.17 (.70, 1.96)	ns	1.21 (.73, 2.02)	ns
Receive Public Assistance	1.25 (.48, 3.31)	ns	1.26 (.48, 3.31)	ns	1.41 (.56, 3.51)	ns	1.43 (.59, 3.46)	ns
Receive Food Stamps	1.31 (.83, 2.07)	ns	1.30 (.82, 2.05)	ns	1.34 (.84, 2.13)	ns	1.28 (.81, 2.04)	ns
Private Insurance Coverage	.65 (.41, 1.02)	ns	.68 (.41, 1.12)	ns	.69 (.42, 1.14)	ns	.67 (.41, 1.82)	ns
Cigarette Use								
Never Use (ref)			–	–	–	–	–	–
Ever Use, Not Past 30-Day			1.57 (.89, 2.77)	ns	1.71 (.97, 3.01)	ns	1.82 (1.02, 3.23)	.042
Past 30-Day Use			2.95 (1.73, 5.03)	<.001	3.43 (2.01, 5.85)	<.001	3.65 (2.11, 6.30)	<.001
Past 30-Day Alcohol Use					.98 (.66, 1.45)	ns	.89 (.60, 1.31)	ns
Screened for Alcohol					2.44 (1.51, 3.97)	<.001	1.48 (.82, 2.67)	ns
Past 30-Day Marijuana Use							1.18 (.85, 1.63)	ns
Screened for Drugs							2.40 (1.41, 4.08)	.002

In multivariable models, having been screened for tobacco use was correlated with a more than 12-fold higher odds of physician advice to quit, controlling for sociodemographics (Model A: OR = 12.60, 95% CI 7.98, 19.88 Table 3) and sociodemographics and cigarette use (Model B: OR = 12.73, 95% CI 8.15, 19.89). In the latter model, Hispanic ethnicity was correlated with significantly lower odds of physician advice to quit smoking (OR = .48, 95% CI .30, .76). Being male (OR = 1.50, 95% CI 1.07, 2.12) and past 30-day cigarette use (OR = 2.95, 95% CI 1.73, 5.03) was correlated with a significantly greater odds of physician advice to quit smoking in this sample.

The relationship between physician tobacco screening and advice to quit was attenuated, but remained strong and significant when alcohol use and screening were added in Model C (OR = 7.12, 95% CI 3.98, 12.74) and marijuana use and drug screening were added in Model D (OR = 6.01, 95% CI 3.35, 10.79). In the final model adjusting for physician screening for tobacco, alcohol, and drug use, physician screening for tobacco use was the strongest correlate of receiving physician advice to quit smoking (OR 6.01; CI 3.35–10.79). In all four models, Hispanic adolescents were significantly less likely to receive advice to quit from their physician compared to non-Hispanic White adolescents.

## Discussion

Although 74% of adolescents sampled in the NSDUH reported visiting a physician in the past year, only 49% were screened for tobacco use by their physician, resulting in an estimated 36% of adolescents being screened for tobacco use at the population level. Only 14.2% of ever and 26.3% of past 30-day cigarette users were screened for and received advice to quit tobacco from their physician. Physician screening for tobacco use was highly correlated with physician advice to quit smoking, after controlling for sociodemographic characteristics, cigarette use, and screening for alcohol use.

The screening and advising rates reported by adolescents in this national study are well below published clinician reports. In a national survey of US primary care physicians, Tong et al. found tobacco screening and advising rates of 97.7% and 94.9%, respectively [19]. Some may argue that this discrepancy is due in part to adolescent recall, but evidence indicates that adolescent recall is more reliable than clinician reports [20, 21]. Another potential contributor to the lower rates is the large non-White population (44.6%) in the study sample. Similar to the disparities documented in adult populations [15, 22–24], we observed lower rates of screening and counseling among non-White adolescents, particularly Hispanic adolescents.

Lower screening rates have been attributed to both individual and institutional factors. Physicians report that brief face-to-face screening and counseling is ineffective, unpleasant, and that they are ill-prepared for such discussions [19, 25, 26]. Additional barriers have been noted when working with smokers in underserved communities including: lack of time, patient unreadiness to change, and inadequate resources for themselves and their patients [27]. Rather than physician-focused strategies to overcome these barriers, arguments have been made for system-level change as a means of fully integrating tobacco prevention and cessation in healthcare delivery [12]. Under system-level recommendations, health care systems would provide physicians with regular trainings, ensure national and local resources are made available to physicians and their patients, and provide feedback to physicians about their tobacco dependence treatment practices [12].

Certain limitations should be considered when interpreting study results. We were unable to make any causal determinations due to the cross-sectional design of the NSDUH, particularly as it pertains to the diminished screening rates. Cigarette use was based on the respondent’s status at the time of the survey and may not accurately reflect their cigarette use status at their last physician visit. Additionally, the self-reported nature of NSDUH may have resulted in over and under-reporting of behaviors.

## Conclusion

Our findings demonstrate low rates of physician tobacco screening among adolescents and an inadequate provision of advice to quit to adolescent smokers, despite a strong relationship between screening and advice to quit. The discrepancy between screening and advising rates reported by adolescents in this national study and in published clinician reports underlines the need for more systematic monitoring and assessment of physician screening and counseling. Regression analyses revealed that use of and screening for other substances attenuated the relationship between screening for tobacco use and advice to quit. Additional research is needed to identify the best practice for screening for multiple substances. Our findings also highlight racial/ethnic disparities in physician screening and advice to quit, particularly among Hispanic adolescents, not previously reported in the adolescent literature. This underscores the need for additional resources specific to these racial/ethnic groups to help facilitate the discussion on tobacco use. Given the critical phase of development adolescence poses in tobacco use and the potential benefit of physician screening and advice to quit during adolescence, further research is needed to enable physicians to have an open dialogue on tobacco use with their adolescent patients.
